# Evaluation of Oxidative Stress Status in Familial Hypercholesterolemia

**DOI:** 10.3390/jcm10245867

**Published:** 2021-12-14

**Authors:** Shiva Ganjali, Reihaneh Keshavarz, Susan Hosseini, Atena Mansouri, Massimo R. Mannarino, Matteo Pirro, Tannaz Jamialahmadi, Amirhossein Sahebkar

**Affiliations:** 1 Department of Medical Biotechnology and Nanotechnology, Mashhad University of Medical Sciences, Mashhad 9177948564, Iran; shivaganjali@gmail.com; 2 Department of Genetics, Faculty of Biological Sciences, Tehran North Branch, Islamic Azad University, Tehran 1651153311, Iran; reihaneh_keshavarz@yahoo.com; 3 Medical Genetics Research Center, Mashhad University of Medical Sciences, Mashhad 9177948564, Iran; su.hosseini.2@gmail.com; 4 Cellular & Molecular Research Center, Birjand University of Medical Sciences, Birjand 9717853577, Iran; mansouri_atena@bums.ac.ir; 5 Biotechnology Research Center, Pharmaceutical Technology Institute, Mashhad University of Medical Sciences, Mashhad 9177948954, Iran; 6 Unit of Internal Medicine, Department of Medicine and Surgery, University of Perugia, 06129 Perugia, Italy; massimo.mannarino@gmail.com (M.R.M.); matteo.pirro@unipg.it (M.P.); 7 Department of Nutrition, Faculty of Medicine, Mashhad University of Medical Sciences, Mashhad 9177948564, Iran; jamiat931@gmail.com; 8 Applied Biomedical Research Center, Mashhad University of Medical Sciences, Mashhad 9177948564, Iran; 9 Department of Biotechnology, School of Pharmacy, Mashhad University of Medical Sciences, Mashhad 9177948954, Iran

**Keywords:** familial hypercholesterolemia, oxidative stress, antioxidant enzymes

## Abstract

Background: Familial hypercholesterolemia (FH) is an autosomal dominant genetic disorder characterizied by elevated levels of circulating low-density lipoprotein cholesterol (LDL-C) which is an important source of substrates to be oxidized by different oxidative agents. Subsequently, the oxidized LDLs (oxLDLs) induce further oxidative reactions in FH patients, which contributes to the development of atherosclerosis and advanced cardiovascular events in these patients. This study aimed to investigate the association of oxidant/antioxidant markers with FH. Methods: This case-control study comprised 18 HoFH, 18 HeFH, and 20 healthy subjects. Oxidant/antioxidant markers including MDA, MPO, thiol, nitric oxide (NO), myeloperoxidase (MPO), glutathione peroxidase (GPx), SOD, and CAT were assessed by colorimetric methods. Prooxidant-antioxidant balance was also measured by pro-oxidant antioxidant balance (PAB) assay. Results: The levels of MDA (*p* < 0.001), MPO activity (*p* < 0.001), thiol (*p* < 0.001), NO (*p* < 0.01), and PAB (*p* < 0.001) were notably higher in HoFH group in comparison with healthy subjects. HeFH group also showed significantly higher levels of thiol (*p* < 0.001) and PAB (*p* < 0.001) when compared to healthy subjects. Elevated levels of MDA (*p* < 0.001) and PAB (*p* < 0.001) were also observed in HoFH relative to HeFH. No significant differences were found between the studied groups in the case of antioxidant enzyme activities. The results of binary logistic regression showed that PAB (OR: 0.979; *p* = 0.033), and MDA (OR: 0.996; *p* = 0.018) levels were inversely associated with HoFH, although, after adjustment for age and LDL-C levels, these associations were diminished. Conclusion: Several oxidant/antioxidant differences were found between FH patients and healthy individuals as well as between HoFH and HeFH patients. These differences might be strongly dependent on plasma LDL-C levels.

## 1. Introduction

Familial hypercholesterolemia (FH) is an autosomal dominant genetic disorder caused by loss-of-function mutations in the gene encoding the low-density lipoprotein receptor (LDLR) leading to the repressing of protein synthesis and its translocation to the cell surface [1]. Further mutations affecting the LDLR binding site on apolipoprotein B (ApoB) and proprotein convertase subtilisin/kexin type 9 (PCSK9) genes, as well as, in signal-transducing adaptor family member 1 which promote LDLR internalization, have also been reported in some cases of FH [2]. All these mutations result in abnormal levels of circulating LDL-cholesterol (LDL-C). FH patients may be either heterozygous (He), with a prevalence of 1 in 250 and plasma LDL-C levels ranging from 5 to 13 mmol/L, or homozygous (Ho), with a frequency of 1 in 1 million and LDL-C levels above 13 mmol/L [3,4]. These patients experience premature cardiovascular (CV) events whose age of appearance is dependent on levels of LDL-C and the possible coexistence of additional CV risk factors [5,6,7,8].

Beyond LDL-C levels and other traditional risk factors, oxidative stress (OS), which means an imbalance between free radicals and antioxidants [9], plays a key role in the initiation and progression of atherosclerosis in FH patients [10,11,12,13,14,15,16]. Hypercholesterolemia in FH patients induces the generation of superoxide radicals that may reduce the activity of endothelial nitric oxide synthase (eNOS) and react with NO; these events result in the reduction of NO bioavailability as an antioxidant and vasodilatory compound with the consequent inflammatory response in the vessel wall [17]. Furthermore, high LDL-C levels may represent an important source of substrates to be oxidized by different oxidative agents, leading to increased formation of lipid peroxidation products like malondialdehyde (MDA), which is also related to atherosclerosis progression in FH patients [18]. Moreover, excess of LDLs induces OS through reduction of antioxidants like glutathione, superoxide dismutase (SOD), and catalase (CAT) as well as increasing the activity of enzymes involved in the production of ROS such as myeloperoxidase (MPO) [12,19].

Therefore, this study aimed to investigate the levels of different OS markers in patients with either HeFH or HoFH and compare them with those in healthy subjects, to find additional markers of increased atherosclerosis risk. 

## 2. Material and Method

### 2.1. Study Population

In this case-control study, 18 HoFH patients as a case group from all over Iran and 18 HeFH patients among family members of HoFH patients and 20 healthy controls have been recruited. Written informed consent was obtained from all participants. The study protocol was approved by the Ethics Committee of the Mashhad University of Medical Sciences (ID: IR.MUMS.PHARMACY.REC.1398.022). The Dutch Lipid Clinic Network Criteria was used to calculate FH scores for patients with suspected FH, including those with elevated cholesterol and/or family history of premature cardiac events. Then DNA testing based on the evaluation of mutations in LDLR, ApoB, and PCSK9 genes by next generation sequencing technique and confirmed by Sanger sequencing method was done for completion of diagnosis. Some of the mutations were previously identified and reported as pathogenic ones according to the ClinVar database, but some of them were novel mutations, the pathogenicity of which was predicted by SIFT database and PolyPhen software. The diagnosis of HoFH was based on the following criteria: (1) genetic confirmation of two mutant alleles at the LDLR, APOB, PCSK9, or LDLRAP1 gene locus OR (2) An untreated LDL-C > 13 mmol/L (500 mg/dL) or treated LDL-C ≥ 8 mmol/L (300 mg/dL), together with either cutaneous OR tendon xanthoma before age 10 years OR untreated elevated LDL-C levels consistent with heterozygous FH in both parents [4]. Moreover, 20 healthy subjects were enrolled who were matched with HeFH group in terms of age and sex. All patients’ information including demographic data, age, gender, and medical history were recorded and blood samples were collected. Instantly, serum was separated by centrifugation of the blood for 20 min at a relative centrifugal force of 1000 (recommended by manufacturer) and then stored at −80 °C prior to analysis.

### 2.2. Lipid Peroxidation Assay

For the determination of lipid peroxidation, the thiobarbituric acid (TBA) assay kit (Kiazist, Iran) was used according to the manufacture’s protocol. This kit is based on the reactivity of MDA, the lipid peroxidation end product, with TBA to produce a chromophore complex which was detected at a wavelength of 532 nm. The MDA concentration was calculated based on a standard curve constructed by MDA standard available in the kit and was reported as nmol/mL of serum.

### 2.3. Total Thiol (–SH) Group Assay

Total thiol (–SH) groups were measured using a colorimetric kit (Kiazist, Iran) in accordance with the manufacturer’s protocol. This kit is based on the reactivity of 5, 5′-dithiobis-(2-nitrobenzoic acid) with reduced sulfhydryl (–SH) groups in the serum and the yellow-generated complex was recorded at 405 nm. The thiol concentration was calculated based on a standard curve constructed by glutathione standard available in the kit and was reported as micro molar (μM).

### 2.4. Enzyme Activity Assay

MPO, glutathione peroxidase (GPx), SOD, and CAT activities were determined using commercial kits (Kiazist; Iran) according to the manufacturer’s protocols. 

MPO activity kit was based on the reduction of hydrogen peroxide to water accompanied by the oxidation of chloride ions to create hypochlorous acid (HOCl), which rapidly reacts with taurine to produce a stable taurine chloramine product. This step readily neutralizes the HOCl, which would otherwise accumulate and inactivate MPO. A catalase-containing stop solution was added to stop MPO catalysis by eliminating hydrogen peroxide. Finally, taurine chloramine reacted with the yellow TNB chromogen probe, with a decrease in color indicating higher MPO activity. Absorbance was measured at 405 nm. The MPO activities in the samples were determined by comparison with the predetermined TNB chromogen standard curve. The MPO activity was reported as mU/well.

GPx activity kit was based on the reduction of hydrogen peroxide to water accompanied by oxidation of glutathione. The changes in absorbance (at 340 nm) were recorded every 1 min for a total of 5 min (Kinetic mode). The GPx activity was reported as mU/mL of serum.

SOD activity kit was based on the ability of SOD to inhibit the conversion of resazurin to resorufin accompanied by reducing superoxide radicals produced by the xanthine/xanthine oxidase system. At the end of reaction resorufin absorbance was recorded at 570 nm. The inhibition rate percentage of resorufin was calculated for each sample, and then it was converted to SOD activity by following formula: (1 U SOD activity = Inhibition rate 50%).

CAT activity kit was based on the neutralization of hydrogen peroxide to water. In this assay, catalase had peroxidase activity in the presence of methanol, then stopped by means of its inhibitor and the generated formaldehyde reacted with Purpald and produced purple color which its absorbance was recorded at 540 nm. Specific activity of catalase is expressed as nmol/min/mL (mU/mL) of serum.

### 2.5. Nitric Oxide Assay

NO was measured using a colorimetric kit (Kiazist, Iran) in accordance with manufacturer’s instruction. This kit is based on the reactivity of Griess reagent with NO in serum and the optical density was recorded at 545 nm while the reference wavelength was 630 nm. The NO concentration was calculated based on a standard curve constructing by nitrate standard available in the kit and was reported as nmol/mL of serum.

### 2.6. Pro-Oxidant Antioxidant Balance (PAB) Assay

The PAB assay is a test to evaluate the oxidants and antioxidants simultaneously in a single test. In this study, PAB was measured according to the previously described method [20]. Briefly, solutions with different proportions (0–100%) of hydrogen peroxide (250 µM) with uric acid (3 mM) (in 10 mM NaOH) were prepared as the standards. To prepare the working solution, 1 mL tetramethylbenzidine (TMB) cation (TMB^+^) solution (containing 400 µL of TMB/DMSO solution, 20 mL of 0.05 M acetate buffer (pH 4.5), 70 µL of 100 mM chloramine T fresh solution, and 25 U of peroxidase enzyme solution) was mixed with 10 mL TMB solution (containing 200 µL of TMB/DMSO solution and 10 mL of 0.05 M acetic acid (pH 5.8)), incubated in dark place for 2 min at room temperature and used directly. 

In each well of 96-well plate, 10 µL of each sample, standard or blank (distilled water), were well mixed with 200 µL working solution. Following the incubation time (12 min in a dark place at 37 °C), 100 µL of 2 N HCl was added to each well and the optical density was evaluated at 450 nm while the reference wavelength was 620 nm or 570 nm. 

The values of PAB assay were expressed in an arbitrary HK (Hamidi-Koliakos) unit based on the percentage of hydrogen peroxide evaluated in standard solution. Finally, the samples PAB values were determined according to the prepared standard curve. 

## 3. Statistical Analysis

All analyses were performed using SPSS software, version 11.5 (Chicago, IL, USA). *p*-values less than 0.05 were considered statistically significant. Continuous variables are presented as mean ± standard error (SE) and differences in variables were distinguished by one-way analysis of variance (ANOVA) and the Tukey multiple comparison post-test, between the studied groups. Categorical variables are presented as percentages and were compared between groups by a Chi square analysis or Fisher’s exact test. The association of LDL-C levels with oxidative stress markers were assessed using Pearson’s correlation coefficients. Binary logistic regression was used to estimate the association between oxidative stress markers with HoFH (Ref: HeFH) after adjustment for age and LDL-C level. Moreover, multinomial logistic regression was used to estimate the association between oxidative stress markers with HoFH and HeFH (Ref: Healthy) after adjustment for age and LDL-C level.

## 4. Results

### 4.1. Baseline Characteristics of Subjects

Of the total number of 56 participants, 18, 18, and 20 were categorized into HoFH, HeFH, and healthy groups, respectively. Groups were not different in the case of gender, while HeFH and healthy groups included older subjects than HoFH group. TC, TG, and LDL-C were significantly lower in healthy and HeFH subjects in comparison with HoFH. Moreover, TC concentration was significantly lower in the healthy group as compared with the HeFH group. HDL-C showed no statistically significant differences between the studied groups (Table 1). All patients in HoFH group had xanthomas and most of them had experienced myocardial infarction (57.9%) and used both statins and ezetimibe (72.2%); none of HeFH showed such signs and just 27.8% of HeFH patients were on statin medication (Table 2).

### 4.2. Comparison of Oxidative Stress Markers between the Studied Groups 

**Figure 1:**
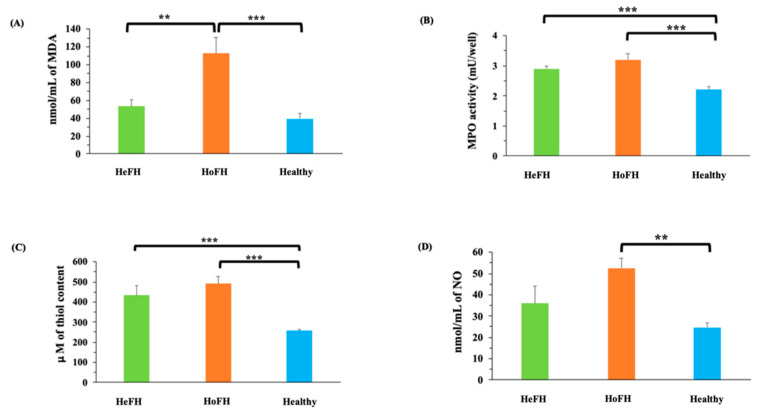
Comparison of (**A**) the MDA levels; (**B**) MPO activity; (**C**) the total thiol content; (**D**) the NO levels of serum between the studied groups. Data are expressed as mean ± SE. ** *p* < 0.01, *** *p* < 0.001. HeFH: Heterozygous familial hypercholesterolemia; HoFH: Homozygous familial hypercholesterolemia; MDA: Malondialdehyde; MPO: Myeloperoxidase; mU/well: milliunit per well; nmol/mL: Nanomol per milliliter NO: Nitric oxide; μM: Micromolar.

**Figure 2:**
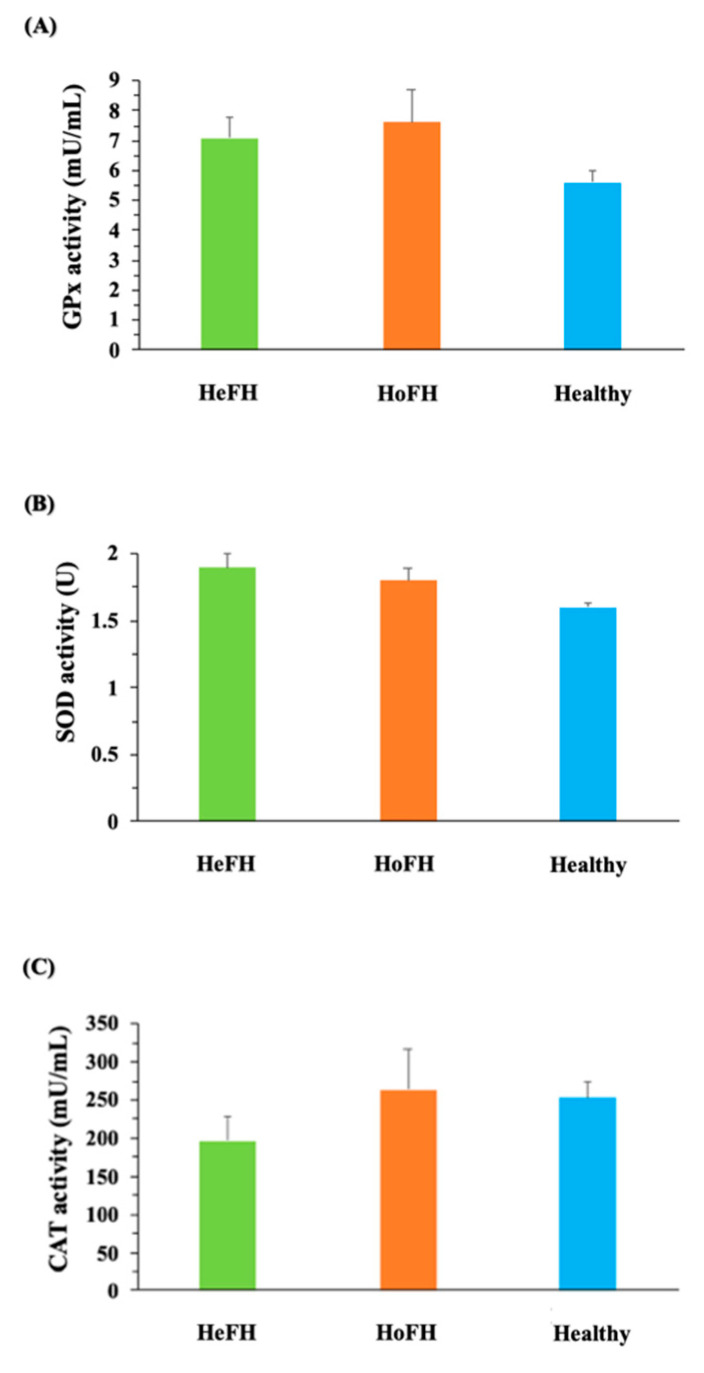
Comparison of the antioxidant enzymes activities between the studied groups. (**A**) GPx activity; (**B**) SOD activity; (**C**) CAT activity. CAT: Catalase; GPx: Glutathione peroxidase; HeFH: Heterozygous familial hypercholesterolemia; HoFH: Homozygous familial hypercholesterolemia; mU/mL: Milliunit per milliliter; SOD: superoxide dismutase; U: Unit.

**Figure 3:**
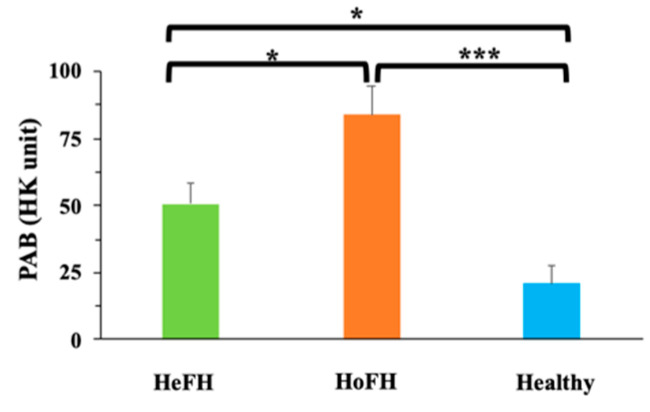
Comparison of the Pro-Oxidant Antioxidant Balance (PAB) between the studied groups. Data are expressed as mean ± SE. * *p* <0.05, *** *p* < 0.001. HeFH: Heterozygous familial hypercholesterolemia; HK unit: Hamidi-Koliakos unit; HoFH: Homozygous familial hypercholesterolemia; PAB: Pro-Oxidant Antioxidant Balance.

Oxidative stress marker analysis illustrated that MDA concentrations were notably higher in HoFH group in comparison with HeFH (*p* < 0.01) and healthy groups (Figure 1A). MPO demonstrated elevated activity in both HoFH and HeFH (*p* < 0.001) groups in comparison with healthy subjects (Figure 1B). Thiol levels were also significantly higher in both HoFH and HeFH (*p* < 0.001) groups in comparison with healthy subjects (Figure 1C). Moreover, a significantly higher level of NO (*p* < 0.01) was observed in HoFH group relative to healthy subjects (Figure 1D). No significant differences in antioxidant enzyme activities (GPx, SOD, and CAT) (Figure 2A, Figure 2B and Figure 2C, respectively) were found between the studied groups. PAB also showed significantly higher levels in both HoFH and HeFH (*p* < 0.001) groups in comparison with healthy subjects, as well as in HoFH group relative to HeFH (Figure 3).

The results of binary logistic regression failed to show any association between antioxidant enzymes activities with HoFH, even after adjustment for age and LDL-C levels. However, PAB (OR: 0.979; *p* = 0.033) and MDA (OR: 0.996; *p* = 0.018) levels were inversely associated with HoFH in this study, though, after adjustment for age and LDL-C levels, these associations were abolished (Table 3).

According to the results of multinomial logistic regression, elevated levels of MDA (OR: 1.049; *p* = 0.001), PAB (OR: 1.058; *p* < 0.001), NO (OR: 1.062; *p* = 0.002) and thiol (OR: 1.020; *p* < 0.001), as well as MPO activity (OR: 2653.722; *p* < 0.001) were significantly associated with HoFH (Ref: healthy), although, after adjustment for age and LDL-C levels, these associations were abolished (Table 4).

In addition, except CAT and SOD activity, strong positive correlations were found between LDL-C levels with all other oxidant/antioxidant markers in the total population, as well as with MPO activity in HoFH group (Table 5).

## 5. Discussion

Elevated plasma LDL-C levels are directly responsible for the increased burden of atherosclerosis and CV risk in patients with FH. However, additional factors along with LDL-C, may contribute to the excess of CV risk in this category of patients [21]. Among these factors, OS appears to trigger atherogenesis in FH patients. In this study, we evaluated OS markers in Iranian FH patients and found that some oxidative and antioxidative markers were significantly higher in FH patients than in healthy controls. In particular, MDA levels, as a lipid peroxidation end-product, were notably higher in HoFH group in comparison with HeFH and healthy groups. The latter results confirm those found in the study by Pirinccioglu et al., in which higher levels of MDA in HoFH in comparison with HeFH and healthy controls were reported [16]. In addition, a significant positive correlation between MDA and LDL-C levels (r = 0.511) was observed in the total population of this study. Similarly, Pirinccioglu et al. found such an association in HoFH patients [16]. Among the mechanisms that may explain the link between hypercholesterolemia and increased OS is the higher MPO activity, an enzyme involved in ROS production and atherosclerotic plaque development. It was suggested that high levels of cholesterol in FH patients could lead to MPO upregulation, so that decreased TC concentration after LDL apheresis is associated with decreased MPO levels [22]. Accordingly, we observed higher MPO activity in both HeFH and HoFH compared to healthy subjects. Our data also showed strong positive correlations between high LDL-C levels and high MPO activity in HoFH patients.

Since increased lipid peroxidation might be a consequence of reduced antioxidant activity, we also measured markers of antioxidant activity like total plasma thiol content and antioxidant enzyme activity. Importantly, our data failed to show any significant difference in antioxidant enzyme activity between the three study groups. Conversely, thiol levels were higher in both HoFH and HeFH than healthy subjects that is in contrast with other studies [23,24] in which thiol concentration was lower in hyperlipidemic patients relative to normolipidemic individuals. A positive correlation between HDL-C and thiol levels was previously reported and it was suggested that the lower level of thiols in hyperlipidemic patients was due to the lower HDL-C concentrations [23]; however, in our study, borderline higher HDL-C levels were observed in both HeFH and HoFH than healthy controls which might lead to the higher thiol levels in these patients. NO is another key antioxidant molecule and its low level was previously reported in the hearts of hypercholesterolemic animals compared to controls [15]. Moreover, it was demonstrated that hypercholesterolemia, by reducing the NO production in endothelial cells was involved in endothelial dysfunction and atherosclerosis [25,26,27]. oxLDL, by reducing the mRNA levels of eNOS, could reduce NO production [28].

In our studied FH patients, despite the elevated levels of LDL-C as a susceptible source to be oxidized, the NO level was surprisingly high in HoFH patients than healthy individuals. Whether the already reported cholesterol-mediated induction of inducible NOS might explain our results should be considered [29]. Moreover, all FH patients were on statin treatment, which exert a pleiotropic antioxidative effects that could have effect on the increasing of NO and thiol content in these patients, compared to healthy subjects.

Finally, the results of PAB assay, which determined the balance between oxidative stress burden and antioxidant defense in a single assay [9], indicated higher values in both HeFH and HoFH groups when compared to healthy individuals, as well as in HoFH patients relative to HeFH. Hence, although antioxidant enzyme activity had not shown any significant alteration between the groups of this study, and high values of thiol and NO were observed in FH patients, the elevated value of PAB in these patients might be due to the enormous oxidant burden in these patients. The disturbance in serum pro-oxidant-antioxidant balance was also reported in subjects with high cholesterol levels [30].

One important limitation in the present study was the lack of background standardization between HoFH and both control groups in terms of age and LDL-C, as OS is closely linked to aging and high LDL-C levels. However, to limit this confounding effect, the association of OS markers with HoFH and HeFH was adjusted for age and LDL-C. Another limitation to be mentioned is the small sample size of our study, which does not allow to draw definitive conclusions. However, it must be remembered that HoFH is a rare and underdiagnosed disease, and ours is the largest population of HoFH patients in which so many markers of OS status have been evaluated at the same time. Nevertheless, confirmation of the present findings, particularly with respect to PAB, would be recommended to be explored in larger populations and multi-center studies, and based on different genetic variant subgroups.

In conclusion, the high levels of PAB and MDA, as well as MPO activity in the serum of FH patients suggest that OS may play a role in the increased cardiovascular risk of FH patients. However, the associations between these markers and FH appeared to be dependent on plasma LDL-C levels. Therefore, according to our results, it is suggested that therapeutic strategies addressing ROS production, antioxidant systems, and prevention of ox-LDL formation may prevent OS and ameliorate atherosclerosis in these patients. Further prospective studies are necessary to confirm the impact of OS in the pathogenesis of atherosclerosis-mediated cardiovascular disease in FH population, as well as to identify newer therapeutic modalities to selectively target oxidative stress in these patients.

## Figures and Tables

**Table 1:** Baseline characteristics of subjects.

Variables		Familial Hypercholesterolemia		Healthy (*n* = 20)	*p*-Value
		HoFH (*n* = 18)	HeFH (*n* = 18)		
Sex	Male	8 (44.4%)	10 (55.6%)	9 (45.0%)	0.751
	Female	10 (55.6%)	8 (44.4%)	11 (55.0%)	
Age (y)		13.10 ± 2.95	31.60 ± 2.00 ^a^	34.50 ± 1.70 ^a^	<0.001
TC (mmol/L)		16.03 ± 1.13	6.7 ± 0.50 ^a^	4.02 ± 0.13 ^a,b^	<0.001
TG (mmol/L)		2.60 ± 0.33	1.23 ± 0.10 ^a^	1.04 ± 0.10 ^a^	0.001
HDL-C (mmol/L)		1.60 ± 0.13	1.73 ± 0.30	1.21 ± 0.10	0.064
LDL-C (mmol/L)		11.01 ± 1.00	4.61 ± 0.50 ^a^	2.41 ± 0.20 ^a^	<0.001

Data are shown as Mean ± SE; ^a^: Significant in comparison with HoFH group; ^b^: Significant in comparison with HeFH group. HDL-C: High-density lipoprotein cholesterol; HeFH: Heterozygous familial hypercholesterolemia; HoFH: Homozygous familial hypercholesterolemia; LDL-C: Low-density lipoprotein cholesterol; mmol/L: Millimoles per liter; TC: Total cholesterol; TG: Triglyceride; y: Year.

**Table 2:** Clinical characteristics of HoFH and HeFH groups.

Variables		Familial Hypercholesterolemia		*p*-Value
		HoFH (*n* = 18)	HeFH (*n* = 18)	
The number of patients with xanthomas symptoms		100%	0%	<0.001
The number of patients with MI history		57.9%	0%	<0.001
Mutation (%)	Previously reported	63.2%	64.7%	0.923
	Novel	36.8%	35.3%	
Mutation type (%)	Missense	52.6%	44.4%	0.331
	Truncated	10.5%	0%	
	Single nucleotide variant	15.8%	27.8%	
	Missense, truncated	5.3%	0%	
	Truncated peptide	5.3%	0%	
*LDLR* position (%)	Exon	87.5%	72.7%	0.370
	Intron	12.5%	27.3%	
Drugs consumption (%)	Only Statin	27.8%	27.8%	0.007
	Statin + Ezetimibe	72.2%	0%	

**Table 3:** Binary logistic regression for oxidative stress markers in relation with HoFH (Ref: HeFH).

Variables	Unadjusted		Adjusted ^#^	
	OR (95% CI)	*p* Value	OR (95% CI)	*p* Value
CAT activity (mU/mL)	0.998 (0.993–1.002)	0.306	0.997 (0.981–1.013)	0.723
GPX activity (mU/mL)	0.965 (0.823–1.133)	0.665	1.051 (0.671–1.645)	0.829
MDA concentration (nmol/mL)	0.996 (0.994–0.995)	0.018	0.007 (0.0–1.6 × 10^112^)	0.971
MPO activity (mU/well)	0.436 (0.141–1.347)	0.149	64.201 (0.235–17,508.817)	0.146
NO concentration (nmol/mL)	0.976 (0.950–1.003)	0.080	1.030 (0.956–1.110)	0.437
SOD activity (U) *	1.713 (0.361–8.134)	0.499	0.989 (0.068–14.465	0.994
Thiol concentration (μM)	0.998 (0.994–1.002)	0.353	1.016 (0.986–1.046)	0.306
PAB (HK unit **)	0.979 (0.959–0.998)	0.033	0.939 (0.835–1.055)	0.290

**#**: Adjusted for age, LDL-C levels. *: 1U SOD Activity = (O2-) Inhibition Rate 50%; **: The percentage of hydrogen peroxide evaluated in standard solution; CI: Confidence interval; CAT: Catalase; GPx: Glutathione peroxidase; HeFH: Heterozygous familial hypercholesterolemia; HK unit: Hamidi-Koliakos unit; HoFH: Homozygous familial hypercholesterolemia; MDA: Malondialdehyde; MPO: Myeloperoxidase; mU/mL: Milliunit per milliliter; mU/well: milliunit per well; nmol/mL: Nanomole per milliliter NO: Nitric oxide; μM: Micromolar; OR: odds ratio; PAB: Pro-Oxidant Antioxidant Balance; SOD: superoxide dismutase; U: Unit.

**Table 4:** Multinomial logistic regression for oxidative stress markers in relation with HoFH and HeFH (Ref: Healthy).

Variables	HoFH				HeFH			
	Unadjusted		Adjusted ^#^		Unadjusted		Adjusted ^#^	
	OR (95% CI)	*p* Value	OR (95% CI)	*p* Value	OR (95% CI)	*p* Value	OR (95% CI)	*p* Value
CAT activity (mU/mL)	1.00 (0.997–1.004)	0.844	0.999 (0.978–1.020)	0.896	0.997 (0.992–1.002)	0.248	0.996 (0.983–1.008)	0.497
GPX activity (mU/mL)	1.216 (0.983–1.505)	0.072	1.032 (0.617–2.744)	0.488	1.166 (0.940–1.447)	0.162	1.369 (0.753–2.490)	0.303
MDA concentration (nmol/mL)	1.049 (1.020–1.080)	0.001	-	0.992	1.018 (0.994–1.043)	0.145	1.010 (0.974–1.047)	0.591
MPO activity (mU/well)	2653.722 (45.857–153,568.335)	<0.001	1.775 (0.001–3781.12)	0.883	1168.432 (21.733–62,828.957)	0.001	113.139 (0.604–21,179.08)	0.077
NO concentration (nmol/mL)	1.062 (1.021–1.104)	0.002	0.971 (0.868–1.086)	0.602	1.032 (0.994–1.072)	0.096	1.00 (0.920–1.088)	0.998
SOD activity (U) *	4.625 (0.642–33.311)	0.128	952.314 (0.552–1,643,155.3)	0.071	9.736 (1.253–75.637)	0.030	947.924 (0.887–1,012,523.6)	0.054
Thiol concentration (uM)	1.020 (1.009–1.032)	<0.001	0.995 (0.963–1.028)	0.761	1.019 (1.007–1.030)	<0.001	1.011 (0.997–1.025)	0.127
PAB (HK unit **)	1.058 (1.026–1.092)	<0.001	1.130 (0.993–1.287)	0.064	1.039 (1.010–1.069)	<0.001	1.061 (1.003–1.123)	0.037

**#**: Adjusted for age, LDL-C levels. *: 1U SOD Activity = (O2-) Inhibition Rate 50%; **: The percentage of hydrogen peroxide evaluated in standard solution; CAT: Catalase; CI: Confidence interval; GPx: Glutathione peroxidase; HeFH: Heterozygous familial hypercholesterolemia; HK unit: Hamidi-Koliakos unit; HoFH: Homozygous familial hypercholesterolemia; MDA: Malondialdehyde; MPO: Myeloperoxidase; mU/mL: Milliunit per milliliter; mU/well: milliunit per well; nmol/mL: Nanomole per milliliter NO: Nitric oxide; μM: Micromolar; OR: odds ratio; PAB: Pro-Oxidant Antioxidant Balance; SOD: superoxide dismutase; U: Unit.

**Table 5:** Correlations between OS markers and LDL-C.

Oxidative Stress Markers	LDL-C							
	HoFH (*n* = 18)		HeFH (*n* = 18)		Healthy (*n* = 20)		Total Population (*n* = 56)	
	r Coefficient	*p*-Value	r Coefficient	*p*-Value	r Coefficient	*p*-Value	r Coefficient	*p*-Value
CAT activity (mU/mL)	−0.318	0.214	−0.212	0.466	−0.116	0.625	−0.086	0.547
GPX activity (mU/mL)	0.287	0.249	0.102	0.718	0.015	0.951	0.335	0.014
MDA concentration (nmol/mL)	0.145	0.592	−0.163	0.562	−0.084	0.726	0.511	<0.001
MPO activity (mU/well)	0.545	0.029	0.252	0.366	0.289	0.217	0.669	<0.001
NO concentration (nmol/mL)	0.141	0.577	0.532	0.05	−0.315	0.188	0.481	<0.001
SOD activity (U) *	0.273	0.29	−0.224	0.422	0.495	0.026	0.147	0.299
Thiol concentration (uM)	0.177	0.497	0.403	0.136	0.075	0.754	0.501	<0.001
PAB (HK unit **)	0.166	0.524	−0.388	0.153	0.135	0.571	0.485	<0.001

*: 1U SOD Activity = (O2-) Inhibition Rate 50%; **: The percentage of hydrogen peroxide evaluated in standard solution; CAT: Catalase; GPx: Glutathione peroxidase; HeFH: Heterozygous familial hypercholesterolemia; HK unit: Hamidi-Koliakos unit; HoFH: Homozygous familial hypercholesterolemia; LDL-C: Low-density lipoprotein cholesterol; MDA: Malondialdehyde; MPO: Myeloperoxidase; mU/mL: Milliunit per milliliter; mU/well: milliunit per well; nmol/mL: Nanomole per milliliter NO: Nitric oxide; μM: Micromolar; PAB: Pro-Oxidant Antioxidant Balance; SOD: superoxide dismutase; U: Unit.

## Data Availability

Data associated with this study are available from the corresponding author on reasonable request.

## References

[1] Benn M., Watts G.F., Tybjærg-Hansen A., Nordestgaard B.G. (2016). Mutations causative of familial hypercholesterolaemia: Screening of 98 098 individuals from the Copenhagen General Population Study estimated a prevalence of 1 in 217. Eur. Heart J..

[2] Raal F.J., Stein E.A., Dufour R., Turner T., Civeira F., Burgess L., Langslet G., Scott R., Olsson A.G., Sullivan D. (2015). PCSK9 inhibition with evolocumab (AMG 145) in heterozygous familial hypercholesterolaemia (RUTHERFORD-2): A randomised, double-blind, placebo-controlled trial. Lancet.

[3] Austin M.A., Hutter C.M., Zimmern R.L., Humphries S.E. (2004). Genetic causes of monogenic heterozygous familial hypercholesterolemia: A HuGE prevalence review. Am. J. Epidemiol..

[4] Cuchel M., Bruckert E., Ginsberg H.N., Raal F.J., Santos R.D., Hegele R.A., Kuivenhoven J.A., Nordestgaard B.G., Descamps O.S., Steinhagen-Thiessen E. (2014). Homozygous familial hypercholesterolaemia: New insights and guidance for clinicians to improve detection and clinical management. A position paper from the Consensus Panel on Familial Hypercholesterolaemia of the European Atherosclerosis Society. Eur. Heart J..

[5] Marks D., Thorogood M., Neil H.A., Humphries S.E. (2003). A review on the diagnosis, natural history, and treatment of familial hypercholesterolaemia. Atherosclerosis.

[6] Turgeon R.D., Barry A.R., Pearson G.J. (2016). Familial hypercholesterolemia: Review of diagnosis, screening, and treatment. Can. Fam. Physician Med. Fam. Can..

[7] de Ferranti S.D., Rodday A.M., Mendelson M.M., Wong J.B., Leslie L.K., Sheldrick R.C. (2016). Prevalence of Familial Hypercholesterolemia in the 1999 to 2012 United States National Health and Nutrition Examination Surveys (NHANES). Circulation.

[8] Ganjali S., Momtazi A.A., Banach M., Kovanen P.T., Stein E.A., Sahebkar A. (2017). HDL abnormalities in familial hypercholesterolemia: Focus on biological functions. Prog. Lipid Res..

[9] Alamdari D.H., Ghayour-Mobarhan M., Tavallaie S., Parizadeh M.R., Moohebati M., Ghafoori F., Kazemi-Bajestani S.M., Paletas K., Pegiou T., Koliakos G. (2008). Prooxidant-antioxidant balance as a new risk factor in patients with angiographically defined coronary artery disease. Clin. Biochem..

[10] Otunola G.A., Oloyede O.B., Oladiji A.T., Afolayan A.J. (2014). Selected spices and their combination modulate hypercholesterolemia-induced oxidative stress in experimental rats. Biol. Res..

[11] Abbas A.M., Sakr H.F. (2013). Simvastatin and vitamin E effects on cardiac and hepatic oxidative stress in rats fed on high fat diet. J. Physiol. Biochem..

[12] Mollazadeh H., Carbone F., Montecucco F., Pirro M., Sahebkar A. (2018). Oxidative burden in familial hypercholesterolemia. J. Cell. Physiol..

[13] Pignatelli P., Menichelli D., Pastori D., Violi F. (2018). Oxidative stress and cardiovascular disease: New insights. Kardiol. Pol..

[14] Li H., Horke S., Förstermann U. (2014). Vascular oxidative stress, nitric oxide and atherosclerosis. Atherosclerosis.

[15] Csonka C., Sárközy M., Pipicz M., Dux L., Csont T. (2016). Modulation of Hypercholesterolemia-Induced Oxidative/Nitrative Stress in the Heart. Oxidative Med. Cell. Longev..

[16] Pirinccioglu A.G., Gökalp D., Pirinccioglu M., Kizil G., Kizil M. (2010). Malondialdehyde (MDA) and protein carbonyl (PCO) levels as biomarkers of oxidative stress in subjects with familial hypercholesterolemia. Clin. Biochem..

[17] Stokes K.Y., Cooper D., Tailor A., Granger D.N. (2002). Hypercholesterolemia promotes inflammation and microvascular dysfunction: Role of nitric oxide and superoxide. Free Radic. Biol. Med..

[18] Rahman T., Hamzan N.S., Mokhsin A., Rahmat R., Ibrahim Z.O., Razali R., Thevarajah M., Nawawi H. (2017). Enhanced status of inflammation and endothelial activation in subjects with familial hypercholesterolaemia and their related unaffected family members: A case control study. Lipids Health Dis..

[19] Dröge W. (2002). Free radicals in the physiological control of cell function. Physiol. Rev..

[20] Ghayour-Mobarhan M., Alamdari D.H., Moohebati M., Sahebkar A., Nematy M., Safarian M., Azimi-Nezhad M., Parizadeh S.M., Tavallaie S., Koliakos G. (2009). Determination of prooxidant--antioxidant balance after acute coronary syndrome using a rapid assay: A pilot study. Angiology.

[21] Bianconi V., Banach M., Pirro M. (2021). Why patients with familial hypercholesterolemia are at high cardiovascular risk? Beyond LDL-C levels. Trends Cardiovasc. Med..

[22] Puntoni M., Sbrana F., Bigazzi F., Minichilli F., Ferdeghini E., Sampietro T. (2011). Myeloperoxidase modulation by LDL apheresis in familial hypercholesterolemia. Lipids Health Dis..

[23] Kinscherf R., Cafaltzis K., Röder F., Hildebrandt W., Edler L., Deigner H.P., Breitkreutz R., Feussner G., Kreuzer J., Werle E. (2003). Cholesterol levels linked to abnormal plasma thiol concentrations and thiol/disulfide redox status in hyperlipidemic subjects. Free Radic. Biol. Med..

[24] Şimşek Ö., Çarlıoğlu A., Alışık M., Edem E., Biçer C.K. (2018). Thiol/Disulfide Balance in Patients with Familial Hypercholesterolemia. Cardiol. Res. Pract..

[25] Feron O., Dessy C., Moniotte S., Desager J.P., Balligand J.L. (1999). Hypercholesterolemia decreases nitric oxide production by promoting the interaction of caveolin and endothelial nitric oxide synthase. J. Clin. Investig..

[26] Pirro M., Schillaci G., Mannarino M.R., Savarese G., Vaudo G., Siepi D., Paltriccia R., Mannarino E. (2007). Effects of rosuvastatin on 3-nitrotyrosine and aortic stiffness in hypercholesterolemia. Nutr. Metab. Cardiovasc. Dis. NMCD.

[27] Pirro M., Schillaci G., Romagno P.F., Mannarino M.R., Bagaglia F., Razzi R., Pasqualini L., Vaudo G., Mannarino E. (2009). Influence of short-term rosuvastatin therapy on endothelial progenitor cells and endothelial function. J. Cardiovasc. Pharmacol. Ther..

[28] Liao J.K., Shin W.S., Lee W.Y., Clark S.L. (1995). Oxidized low-density lipoprotein decreases the expression of endothelial nitric oxide synthase. J. Biol. Chem..

[29] Kim J.W., Kang K.W., Oh G.T., Song J., Kim N.D., Pak Y.K. (2002). Induction of hepatic inducible nitric oxide synthase by cholesterol in vivo and in vitro. Exp. Mol. Med..

[30] Balkan J., Doğru-Abbasoğlu S., Aykaç-Toker G., Uysal M. (2004). Serum pro-oxidant-antioxidant balance and low-density lipoprotein oxidation in healthy subjects with different cholesterol levels. Clin. Exp. Med..

